# Human breast adipose tissue: characterization of factors that change during tumor progression in human breast cancer

**DOI:** 10.1186/s13046-017-0494-4

**Published:** 2017-02-07

**Authors:** Sabrina Johanna Fletcher, Paula Alejandra Sacca, Mercedes Pistone-Creydt, Federico Andrés Coló, María Florencia Serra, Flavia Eliana Santino, Corina Verónica Sasso, Constanza Matilde Lopez-Fontana, Rubén Walter Carón, Juan Carlos Calvo, Virginia Pistone-Creydt

**Affiliations:** 1 Laboratory of Proteoglycans Chemistry and Extracellular Matrix, Institute of Biology and Experimental Medicine (IByME), National Scientific and Technical Research Council (CONICET), Buenos Aires, Argentina; 2 Gynecological Surgery and Breast Pathology, Oncology Institute “Alexander Fleming”, Buenos Aires, Argentina; 3 Laboratory of Hormones and Cancer Biology, Institute of Medicine and Experimental Biology of Cuyo (IMBECU), National Scientific and Technical Research Council (CONICET), Mendoza, Argentina; 4 Department of Biological Chemistry, School of Exact and Natural Sciences, University of Buenos Aires, Buenos Aires, Argentina; 5 Laboratory of Hormones and Cancer Biology, Institute of Medicine and Experimental Biology of Cuyo, Av. Ruiz Leal w/n, General San Martin Park, Mendoza, CP5500 Argentina

**Keywords:** Human breast adipose tissue, Breast epithelial cells, Breast cancer, Epithelial-stromal interactions

## Abstract

**Background:**

Adipose microenvironment is involved in signaling pathways that influence breast cancer. We aim to characterize factors that are modified: 1) in tumor and non tumor human breast epithelial cell lines when incubated with conditioned media (CMs) from human breast cancer adipose tissue explants (hATT) or normal breast adipose tissue explants (hATN); 2) in hATN-CMs *vs* hATT-CMs; 3) in the tumor associated adipocytes *vs*. non tumor associated adipocytes.

**Methods:**

We used hATN or hATT- CMs on tumor and non-tumor breast cancer cell lines. We evaluated changes in versican, CD44, ADAMTS1 and Adipo R1 expression on cell lines or in the different CMs. In addition we evaluated changes in the morphology and expression of these factors in slices of the different adipose tissues. The statistical significance between different experimental conditions was evaluated by one-way ANOVA. Tukey’s *post-hoc* tests were performed within each individual treatment.

**Results:**

hATT-CMs increase versican, CD44, ADAMTS1 and Adipo R1 expression in breast cancer epithelial cells. Furthermore, hATT-CMs present higher levels of versican expression compared to hATN-CMs. In addition, we observed a loss of effect in cellular migration when we pre-incubated hATT-CMs with chondroitinase ABC, which cleaves GAGs chains bound to the versican core protein, thus losing the ability to bind to CD44. Adipocytes associated with the invasive front are reduced in size compared to adipocytes that are farther away. Also, hATT adipocytes express significantly higher amounts of versican, CD44 and Adipo R1, and significantly lower amounts of adiponectin and perilipin, unlike hATN adipocytes.

**Conclusions:**

We conclude that hATT secrete a different set of proteins compared to hATN. Furthermore, versican, a proteoglycan that is overexpressed in hATT-CMs compared to hATN-CMs, might be involved in the tumorogenic behavior observed in both cell lines employed. In addition, we may conclude that adipocytes from the tumor microenvironment show a less differentiated state than adipocytes from normal microenvironment. This would indicate a loss of normal functions in mature adipocytes (such as energy storage), in support of others that might favor tumor growth.

## Background

In addition to epigenetic and genetic changes that occur within epithelial cells, research in the last few years has shown that tumor progression also depends on the dialog between tumor epithelial cells and surrounding stromal cells [1]. Among the different cell types that surround breast tumor epithelial cells, the most abundant are those that compose breast adipose tissue, mainly mature adipocytes, preadipocytes and adipose tissue derived stem cells (ASC). Adipose tissue is a bioactive endocrine organ [2, 3] that secretes soluble factors and contributes significantly to the composition of the extracellular matrix (ECM). Visceral adipose tissue is different from peripheral adipose tissue since it produces a set of unique cytokines and growth factors [4].

The importance of stromal adipocytes during the development of the normal mammary gland has been recently demonstrated [5]. In addition, in the past few years several groups have demonstrated the importance of the interaction that is established between tumor cells and stromal adipocytes within the invasive front [6, 7]. Cells seem to be able to modify adipocyte phenotype, which in turn, would stimulate tumor aggressive behavior and local invasion [8]. The involvement of factors such as leptin, adiponectin, COLVI, IL-6, HGF and VEGF has been suggested, although there is still a lack of results in human tissues that might confirm their role as well as the potential involvement of new components. Recent studies have demonstrated that growth factors and cytokines secreted by adipose tissue have a significant impact on the progression of several diseases, including breast cancer [3, 8–12]. Statistics show that breast cancer has one of the highest mortality rates around the world. For women in Argentina it is the primary cause of death by cancer (20.1/100,000 women, National Cancer Institute, Argentina, January 2016). Therefore, from a clinical point of view, it is highly relevant to study the involvement of breast adipose tissue in breast cancer development. In particular, it would be relevant to characterize factors secreted by these adipose cells that could modify several aspects of normal and tumor breast epithelial cell lines.

In this study, we focused on ECM proteins, adipokines and their receptors. Versican, a chondroitin sulphate proteoglycan is involved in intracellular signaling and in connecting the cell with the ECM. Increased levels of this proteoglycan have been found in some tumors [13, 14], suggesting that an increase in versican might contribute to tumor progression. Several works have shown that versican seems to be involved in diverse cell functions such as adhesion, migration and proliferation [15].

CD44, which is a transmembrane glycoprotein that belongs to the family of cell adhesion molecules (CAMs), regulates cell-cell and cell-matrix interactions. Therefore, it plays a key role in cell adhesion and migration. It is also a marker for stem cells in several cell types including epithelial cells and adipocytes [16, 17]. Some works have associated increased levels of CD44 with poor prognosis in cancer development [18, 19].

During the phase of tumor progression, degradation of different local components of the ECM, followed by *de novo* production of matrix proteins, seem to be fundamental prerequisites for metastatic development. ADAMTS (a disintegrin and metalloproteinase with thrombospondin motifs) in particular are a group of proteases capable of proteoglycan cleavage and ECM degradation. Some works have described the differential expression of ADAMTS in breast cancer, observing a deregulation of ADAMTS [20].

Adiponectin and leptin are the two main adipokines secreted by adipocytes. Their role on breast cancer has been extensively studied. Most research show that leptin and adiponectin have opposite effects on cancer development, being leptin pro-tumorigenic and pro-angiogenic [21–23]. However results about these adipokines and their receptors (Adipo R1, Adipo R2 and ObR) have sometimes been contradictory and thus not conclusive [24].

Models used to study the dialogue between adipose tissue and breast cancer include preadipocyte immortalized cell lines, animal models and 3D culture systems. We have recently shown that conditioned media (CMs) from human breast cancer adipose tissue explants (hATT) regulate proliferation, adhesion and migration of breast cancer epithelial cell lines, as opposed to CMs from normal breast adipose tissue explants (hATN) [12]. In the present work we aim to characterize factors that are modified in tumor and non tumor human breast epithelial cell lines when incubated with hATT- or hATN-CMs, and are possibly involved in the regulation of cell proliferation, adhesion and migration. Specifically, we evaluated changes in the expression of versican, CD44, ADAMTS1, and Adipo R1. In addition, we evaluated the levels of versican and ADAMTS1 in hATN-CMs *vs* their expression in hATT-CMs. Previously, we have shown that hATT-CMs increase cell migration. In the present work, we found that this effect is lost when hATT-CMs are pre-treated with Chondroitinase ABC. Finally, we observed changes in the phenotype of the tumor associated adipocytes compared to non tumor associated adipocytes; and evaluated by means of immunohistochemistry the expression of versican, adiponectin, AdipoR1, CD44 and perilipin (a marker for mature differentiated adipocytes) [25] in hATT and hATN.

The identification of these factors, both in adipose tissue and epithelial cells, and the study of their possible involvement in the regulation of tumor progression, might help develop new strategies to prevent and/or treat breast cancer.

## Methods

### Reagents

Reagents were purchased from Sigma Chemical Co (St. Louis, MO, USA), tissue culture flasks, dishes, and multi-well plates were from Falcon Orange Scientific (Graignette Business Park, Belgium), culture media and supplements for both tissue and cell lines were from Gibco BRL (Carlsbad, CA, USA).

### Sample collection and handling

For the experiments we used fragments of adipose tissue from both tumoral (hATT, *n* = 22) and normal breasts (hATN, *n* = 16). These fragments were embedded in paraffin. hATT tissue samples were obtained from estrogen and/or progesterone receptor positive, stage GH2, infiltrating ductal carcinomas. None of the patients had received chemotherapy or radiotherapy treatment. hATN tissue samples were obtained from plastic surgeries performed for aesthetic reasons (breast reduction). Al samples were processed within 2 h under a sterile laminar flow hood. At arrival, tissue was transferred to a Petri dish and was extensively washed with 50 ml ice PBS supplemented with gentamicin (50 μg/ml). Thereafter, tissues were transferred to a 50 ml centrifuge tube containing 45 ml phosphate buffer saline solution (PBS) at 37 °C and gently shaken for 5 min and centrifuged for 1 min at 277 x g at RT to remove red blood cells and debris. Tissue was then removed from the tube and weighed. hATT or hATN tissue was placed in a culture flask with M199 culture medium (Invitrogen™) (1 g tissue/10 ml M199) supplemented with gentamicin (50 μg/ml) and incubated for 1 h at 37 °C in 5% CO_2_. After this time, medium was removed and replaced with fresh medium. After 24 h CM was collected, centrifuged, filtrated and aliquoted into 1 ml fractions and immediately stored at -80C, until its use. M199 medium was used as Control-CM. All patients gave their written consent. Samples (tumor and normal) were collected following the approval of the Ethics Committee of the IBYME [12].

### Culture of tumor and non-tumor breast epithelial cell lines

Immortalized epithelial cell lines from human tumor (MCF-7 or IBH7) and non tumor (MCF10-A and HBL100) breasts were used. MCF-7, MCF-10A and HBL100 were obtained from the American Type Culture Collection (ATCC, Rockville, MD, USA), whereas IBH-7 is a line immortalized from a primary breast tumor by the laboratory of Dr. Isabel Luthy [26, 27]. MCF10-A cell line cultures were grown in DMEM-F12 medium supplemented with 10% bovine fetal serum (BFS), insulin (2 μg/ml), cortisol (0.5 μg/ml) and EGF (20 ng/ml) and antibiotics; while the remaining cell line cultures were grown in DMEM-F12 supplemented with 10% BFS and insulin (2 μg/ml), and antibiotics. All cultures were kept at 37 °C in a 5% CO_2_ atm.

### Breast epithelial cell proliferation assay

Tumor (3x10^3^ MCF-7 or IBH-7 cells/well) and non-tumor (5x10^3^ MCF-10A or HBL100 cells/well) human breast epithelial cell lines were incubated on 96-well plates with complete DMEM-F12 for 24 h. Then, the four cell lines were treated with hATN-, hATT- or control-CMs for an additional 48 h, at 50% CM (50% hATN-, hATT- or control-CMs and 50% DMEM-F12 medium) or 75% CM (75% hATN-, hATT- or control-CMs and 25% DMEM-F12 medium). The number of viable cells was determined by a commercial colorimetric kit (Cell titer 96 AQueous One Solution Cell Proliferation Assay, MTS). Results are expressed as percentage of color intensity and normalized to cells grown in control-CMs.

### Preparation of cell lysates from breast epithelial cells after incubation with hATN-CMs or hATT-CMs

MCF-10A, HBL100, MCF-7 and IBH-7 cells were seeded in six-well plates in DMEM-F12 complete medium. When cells reached 75–80% confluence, the medium was aspirated and cells were washed twice with PBS. Then, cells were incubated at 37 °C for 24 h either with hATT-, hATN-, or control-CMs (50% CM, 50% DMEM-F12). Cells were lysed with Ripa buffer, pelleted by centrifugation at 4 °C and stored at −80 °C.

### Immunofluorescence

Tumor (MCF-7 or IBH-7) and non-tumor (MCF-10A and HBL100) were seeded on Lab Tek Chamber Slide system until they reached 50–60% confluence. They were then incubated with hATT-, hATN-, or control-CMs for 24 h (50% CM, 50% DMEM-F12). An additional control without primary antibody was performed as antibody specificity control. Cells were fixed with formaldehyde, washed, blocked and permeabilized (PBS-BSA 0.2%-Triton X100 0.3% for 30 min). They were then incubated with 100 μl of anti-versican primary antibody (Santa Cruz, USA) at 4 °C. In order to increase specificity, primary antibody unspecific activity was blocked with goat serum. Cells were then washed and incubated with the second antibody conjugated to FITC (Sigma) for 1 h. Finally they were incubated for 10 min with propidium iodide to visualize nuclei. Cell localization (nuclear or cytoplasmic) was visualized, and fluorescence intensity was quantified per cell using ImageJ 1.42q software available at the NIH site (http://rsb.info.nih.gov/ij).

### Western blot analysis

In order to evaluate protein expression levels, Western blots were performed. Versican, CD44, ADAMTS1 and Adipo R1 were measured after incubation of the epithelial cell lines with the different CMs obtained. In order to lyse cells, Ripa buffer was used (Tris 10 mM pH 7,5; NaCl 150 mM; sodium vanadate 2 mM; sodium deoxycholate; SDS 0,1%; igepal 1%; protease inhibitors). In addition, versican and ADAMTS1 expression was measured in CMs-hATT and CMs-hATN. Total protein in samples was quantified by Bradford method. Proteins were separated in a SDS-PAGE 10% gel, and electrotransferred to a nitrocellulose membrane (Amersham). The membrane was later blocked with bovine serum albumin (Sigma-Aldrich, 0055 K) and then incubated with the different antibodies ON at 4 °C. The membranes were later washed, and incubated with proper secondary antibodies conjugated to HRP. Antibody complexes were visualized by means of chemiluminescence (ECL; GE Helathcare). Band density was measured by means of SCION Image^TM^ software (Scion Corporation, Maryland, USA). In the cell extracts, β-actin level in samples was used to determine that equal quantities of proteins were loaded in the gel.. The assay for the CMs-hATT and CMs-hATN was done by loading equal volumes of each CM.

### Breast epithelial cell migration assays (“wound-healing”)

The effect of hATN- or hATT-CMs, with or without Chondroitinase ABC, on the motility of tumor breast epithelial cell lines was evaluated by wound-healing assays.

MCF-7 and IBH-7 cells were grown on 96-well plates with complete DMEM-F12 until cells reached 100% confluence. After, cell monolayers were scratched with a 200 μl pipette tip, washed twice with PBS and hATN-, hATT- or control-CMs were added. CMs were pre-treated with Chondroitinase ABC (final concentration 80 mU/ml) (Sigma C2905) or PBS for 1 h at 37 °C. Images at time zero (0 h) were captured to record the initial width of the wounds, and the recovery of the wounded monolayers due to cell migration toward the denuded area was evaluated at 6 h. The images were captured using an inverted phase-contrast microscope (Olympus CKX-41; 4x objective). A quantitative analysis of the wound closure was measured by Java Image J (NIH, Bethesda, MD, USA) software using the freehand selection mode and determined the relative wound closure respect to control of the wounded area after 6 h.

### H&E staining

Tissues (hATT and hATN) were fixed in 4% formaldehyde and embedded in paraffin. They were afterwards cut into sections of 3 *μ*m thickness with a microtome, deparaffinized and stained with hematoxylin-eosin (H&E). Images were taken with a Nikon Eclipse E200 Microscope fitted with a digital still camera Micrometric SE Premium (Nikon Corp., Japan) at 100x magnification. Adipocyte area quantification (measuring adipocyte perimeter) in the three tissue types was performed in 8–10 fields of each preparation as mentioned above. In particular, we compared tissues obtained from hATN with two different areas of hATT: adipose tissue attached to the primary tumor; and adipose tissue obtained 2 cm away from the tumor.

### Immunohistochemistry

Serial cuts were performed on the same tissue samples used for H&E staining. Versican, adiponectin, Adipo R1, CD44, and perilipin expression were studied by means of immunohistochemistry. Briefly, hATT and hATN microtome slides were first deparaffinized, and then a heat-mediated antigen retrieval, endogenous peroxidase blocking and nonspecific tissue blocking were performed. Slides were then incubated with the different primary antibodies at 4 °C, and after that with an anti-rabbit biotinylated secondary IgG antibody. Finally, slides were incubated with peroxidase-conjugated streptavidin. Peroxidase reaction was performed with chromogen 3,3′-diaminobenzidine (DAB) (DAKO LSAB + Kit, HRP). Hematoxylin counter stain was performed. Serial cuts incubated in the absence of primary antibody were used as negative controls. Images were taken with a Nikon Eclipse E200 Microscope fitted with a Micrometric SE Premium (Nikon Corp., Japan) digital still camera at 100x and 400x magnifications. DAB staining quantification in the three tissue types was performed in 8–10 fields of each preparation as mentioned above.

### Statistical analysis

The statistical significance between different experimental conditions was evaluated by one-way ANOVA. Tukey’s *post-hoc* tests were performed within each individual treatment. The results are presented as mean ± SEM. Results were considered significant at *p* < 0.05.

## Results

### hATT-CMs increase proliferation of tumor and non-tumor epithelial cell lines in a dose-dependent manner

**Fig. 1:**
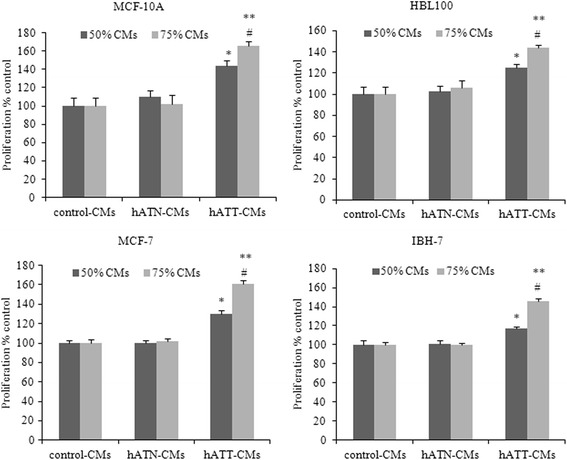
*Effect of CMs from hATN and hATT on proliferation of MCF-10A, HBL100, MCF-7 and IBH-7 cells.* MCF-10A, HBL100, MCF-7 and IBH-7 cells were incubated with hATN- (*n* = 10), hATT- (*n* = 12) or control-CMs 48 h, at 50% or 75% CM. Proliferation was measured by MTS assays. Data are shown as the mean ± SEM (*n* = 5–7 experiments by triplicate). *p < 0.001 hATT-CMs *vs.* hATN-CMs or control-CMs (50% CMs); ^#^ *p* < 0.001 hATT-CMs *vs.* hATN-CMs or control-CMs (75% CMs); **p < 0.05 50% hATT-CMs *vs.* 75% hATT-CMs

We evaluated the effect of human adipose tissue CMs obtained from tumor (hATT) and normal (hATN) breasts in the proliferation of MCF-10A, HBL100, MCF-7 and IBH-7 cells. After 48 h of incubation, hATT-CMs significantly increased proliferation of both tumor (MCF-7 and IBH-7) and non-tumor (MCF-10A, HBL100) epithelial cells, compared to the effect of hATN-CMs and control -CMs (*p* < 0.001) (Fig. 1). In addition, hATN-CMs did not modify the proliferation of any of the four cell lines compared to control-CMs. A significant increase of proliferation was observed when tumor cell lines were incubated with 75% of the hATT-CMs, compared to incubation with 50% of these same CMs (*p* < 0.05) (Fig. 1).

These results are in accordance with previous ones by our group [12], and suggest the presence of soluble factors in hATT-CMs (either absent or present at lower concentrations, in hATN-CMs) that would stimulate the proliferation of both tumor and non-tumor epithelial cells. The characterization of soluble factors in both CMs will help to elucidate this question.

### hATT-CMs increase versican expression in breast cancer epithelial cells

Versican proteoglycan can participate in the regulation of cell motility, growth, differentiation and angiogenesis. Its cell location has been described to depend on growth conditions and cell type. Preliminary results had shown presence of versican in the cell lines employed. Nevertheless, we modified the antibody protocol to confirm the specificity of the antibody used to recognize versican (primary antibody unspecific activity was blocked with goat serum).

**Fig. 2:**
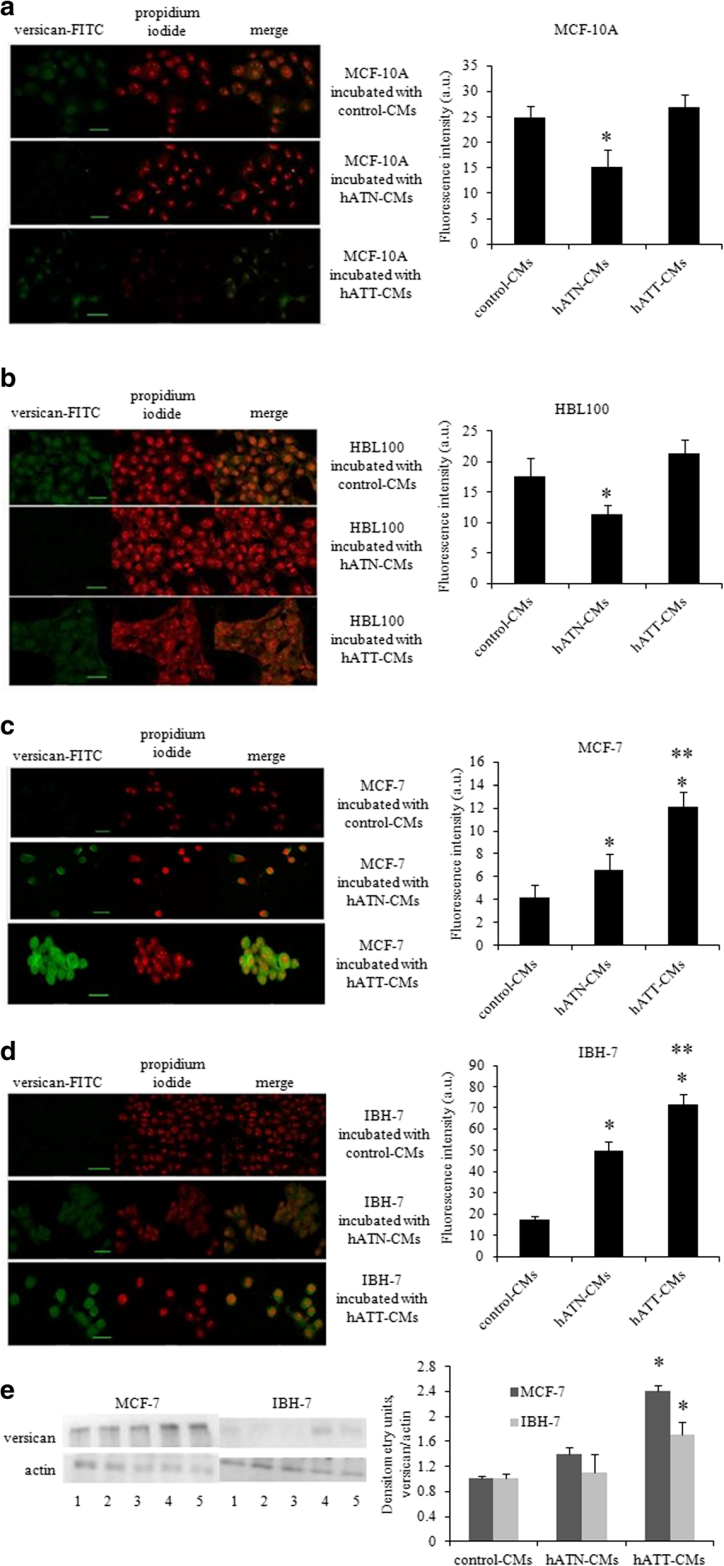
*Effect of CMs from hATN and hATT in localization/expression of versican in MCF-10A, HBL100, MCF-7 and IBH-7 cells.* **a**-**d** MCF-10A, HBL100, MCF-7 and IBH-7 cells were seeded on Lab Tek Chamber Slide system and allowed to grow for 24 h. They were then incubated for 24 h with the different CMs and versican immunodetection was performed. Fluorescence was observed with confocal microscopy. Representative images are shown (scale bar: 20 μm). **a** MCF-10A, **b** HBL100, **c** MCF-7 and **d** IBH-7. Histograms show mean ± SEM of total cell fluorescence. *Green*: versican; *red*: nuclei (a.u.: arbitrary units) (*n* = 3, duplicate experiments) *p < 0.001 hATT-CMs and/or hATN-CMs *vs.* control-CMs; **p < 0.01*.* hATT-CMs *vs* hATN-CMs. **e** MCF-7 and IBH-7 were grown on 6 well plates, incubated for 24 h with the different CMs and cells were lysed. Versican expression was measured by Western blot. 1- cells incubated with control-CMs; 2 and 3- cells incubated with hATN-CMs; 4 and 5- cells incubated with hATT-CMs. β-actin was used as internal control. Images were analyzed by densitometry. Bars show mean ± SEM of three independent experiments. *p < 0.05 tumor cells incubated with hATT-CMs *vs.* hATN-CMs or control-CMs

We found a nuclear and cytoplasmic localization of versican in the four cell lines used, being more intense in MCF-10A and HBL100 (Fig. 2a–d). In addition, we observed a significant increase in total fluorescence intensity in MCF-7 and IBH-7 cells incubated with hATT-CMs compared to the expression found with hATN-CMs and control-CMs (Fig. 2c and d). Furthermore, we found that when non-tumor cells, MCF10A and HBL100, are treated with hATN-CMs, there is actually a decrease in fluorescence intensity for versican. This decrease of intracellular versican could be due to an increase of versican release to the ECM from both non-tumoral cells or reduce expression of versican.

The increase in the expression of versican was also observed by Western-blot, in cell lysates of MCF-7 and IBH-7 incubated with hATT-CMs compared to the expression found with hATN-CMs and control-CMs (Fig. 2e).

### hATT-CMs increase CD44, ADAMTS1 and Adipo R1 expression in breast tumor cancer epithelial cells

We evaluated possible changes in the expression of these proteins in the different cell lines incubated with the CMs (hATT-, hATN- or control-CMs).

**Fig. 3:**
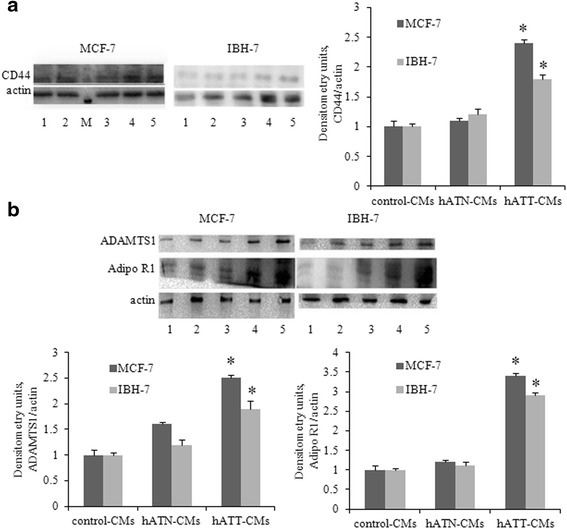
*Effect of CMs from hATN and hATT on: CD44* (**a**); *ADAMTS1 and Adipo R1* (**b**) *expression was evaluated in MCF-7 and IBH-7 cells.* MCF-7 and IBH-7 cells were grown on 6 well plates, incubated for 24 h with the different CMs and then lysed. Expression of the different proteins was measured by Western blot. 1- Cells incubated with control-CMs; 2 and 3- cells incubated with hATN-CMs; 4 and 5- cells incubated with hATT-CMs. β-actin was used as internal control. Images were analyzed by densitometry. Histograms show mean ± SEM of three/five independent experiments. *p < 0.05 tumor cells incubated with hATT-CMs *vs.* hATN-CMs or control-CMs

We observed an increase in CD44 expression in tumor cells MCF-7 and IBH-7 incubated with hATT-CMs compared to the value observed with hATN- or control-CMs (Fig. 3a).

ADAMTS1 is a member of a group of peptidases (different from proteases) that are able to cleave proteoglycans and degrade the ECM. Our results indicate an increased expression of the 87 kDa form of ADAMTS1 in MCF-7 and IBH-7 tumor cells incubated with hATT-CMs vs. hATN- or control-CMs (Fig. 3b).

Adipose tissue produces adiponectin, which has been proposed to be involved in breast cancer regulation through its membrane receptors. Our results indicate an increased expression of Adipo R1 in MCF-7 and IBH-7 tumor cells incubated with hATT-CMs *vs.* hATN- or control-CMs (Fig. 3b).

No significant differences were found with any treatment in non-tumor cells MCF-10A and HBL100.

### hATT-CMs present increased versican expression, and no significant changes in ADAMTS1 expression, compared to hATN-CMs

We evaluated the expression of the proteoglycan versican and metalloproteinase ADAMTS1 in hATT- and hATN-CMs. Previous research has described the differential expression of ADAMTS1 in breast cancer and a deregulation of ADAMTS1 in this cancer type.

**Fig. 4:**
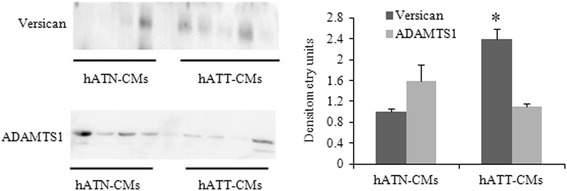
*Versican and ADAMTS1 in hATT- and hATN-CMs.* Versican and ADAMTS1 expression was evaluated by Western blot. Images were analyzed by densitometry. Histograms show mean ± SEM of two independent experiments (*n* = 9). *p < 0.05 hATT-CMs *vs.* hATN-CMs

Our results indicate an increased expression of versican in hATT-CMs compared to hATN-CMs. We found that the 65kDA form of ADAMTS1 was present both in hATT-CMs and hATN-CMs. Although no significant differences were found in this protease, a tendency to be augmented in the hATN-CMs was seen (Fig. 4). These results indicate that adipose tissue present in tumor breasts has increased versican secretion, compared to adipose tissue from normal breasts.

### Chondroitinase ABC treatment of hATT-CMs eliminates their effect on cell migration of breast cancer epithelial cells

**Fig. 5:**
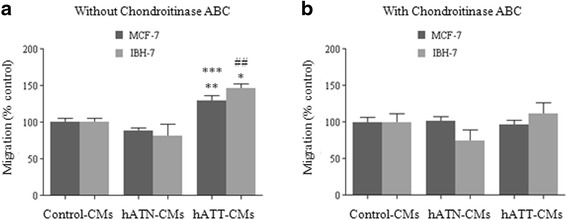
*Involvement of chondroitin sulfate proteoglycans on the effect of CMs from hATN and hATT on migration of MCF-7 and IBH-7 cells*. Wound healing assay (**a**,**b**). MCF-7 and IBH-7 cells were grown until they reached 100% confluence. After, cells were wounded and hATN-, hATT- or control-CMs were added. CMs were pre-treated with Chondroitinase ABC (**b**) or PBS (**a**) for 1 h at 37 °C prior to the incubation of the cells. Images were captured at the wound instant and after 6 h. Histograms show the closure after 6 h relative to the control and was plotted as mean ± SEM of two/three independent experiments (*n* = 7 by duplicate). *p < 0.05 hATT-CMs *vs.* control-CMs; **p < 0.01 hATT-CMs *vs.* control-CMs; ***p < 0.001 hATT-CMs *vs.* hATN-CMs; ##*p* < 0.01 hATT-CMs *vs.* hATN-CMs

We performed “wound healing” assays to evaluate the effect on cell migration of MCF-7 and IBH-7 incubated with control-, hATN- and hATT-CMs pre-treated (or not) with chondroitinase ABC, an enzyme that degrades versican’s GAG chains, therefore preventing effective interaction between versican and tumor cells (via CD44). CMs were pre-treated with Chondroitinase ABC (or PBS) for 1 h at 37°c prior to the incubation of the cells. We observed that in both cell lines hATT-CMs significantly increased cell migration *vs. *control- and hATN-CMs (Fig. 5a). Interestingly, this effect is lost when CMs are pre-treated with chondroitinase ABC (Fig. 5b).

### Adipocytes associated with the invasive front are reduced in size compared to adipocytes that are farther away

**Fig. 6:**
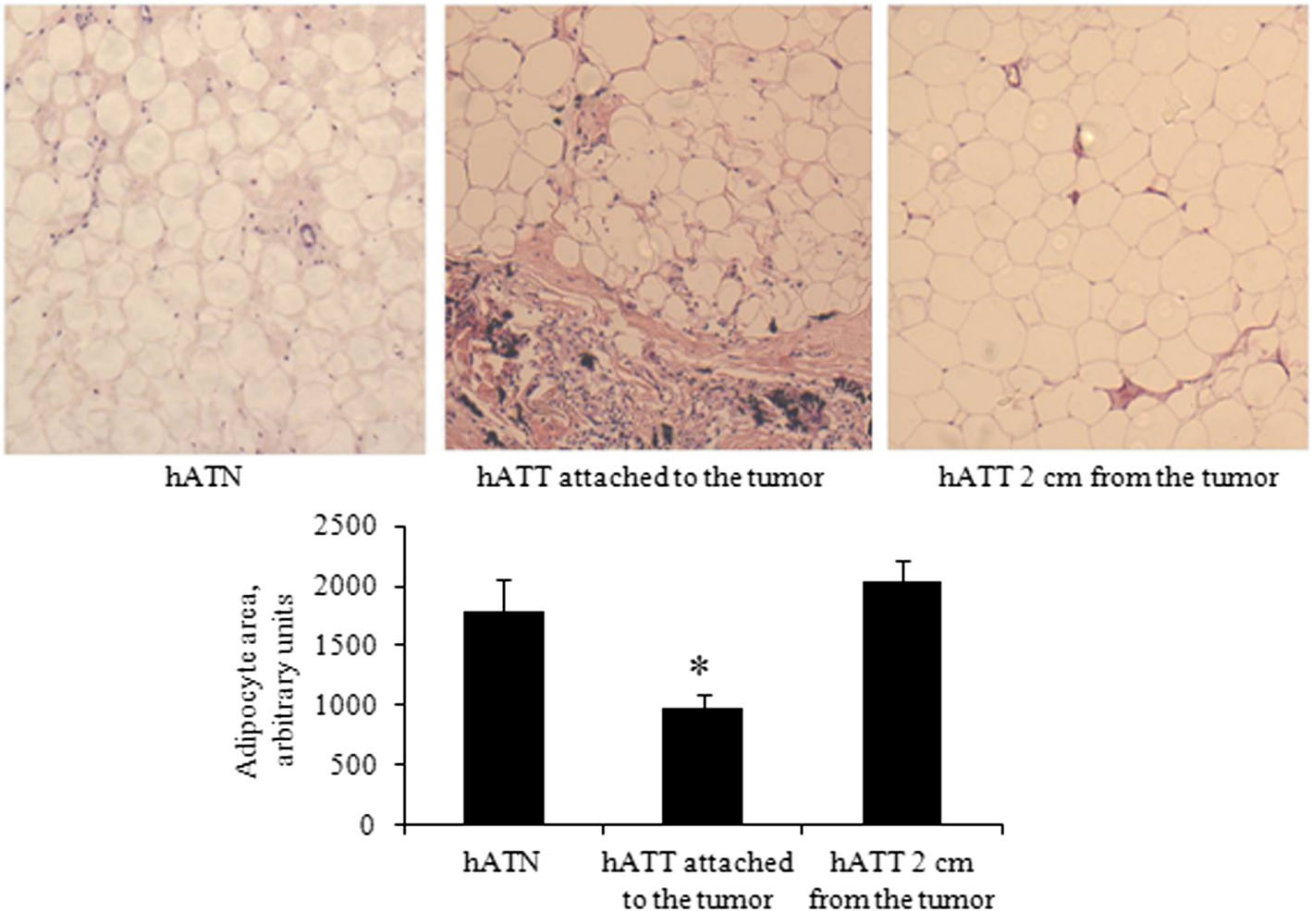
*Microscopic evaluation of different breast adipose tissues.* Slides from: 1) adipose tissue samples from normal breasts of hATN; 2) adipose tissue samples of tumor breast attached to the tumor of hATT; and 3) adipose tissue sample of tumor breast aproximately 2 cm away from the tumor of hATT. Adipose tissue fragments were cut in paraffin, stained with H&E, and observed under light microscope. Adipocyte size was quantified with Image J software (NIH). Histograms show mean ± SEM of two independent experiments. *p < 0.05 hATT attached to the tumor *vs.* hATT 2 cm away from the tumor and hATN. Magnification: 100X

We evaluated changes in the histoarchitecture of different breast adipose tissues. In particular, we compared: 1) adipose tissue from normal breasts; 2) adipose tissue attached to the tumor; and 3) adipose tissue 2 cm away from the tumor. We observed that the characteristics of the connective tissue were modified as a response to local invasion by tumor cells. Specifically, tumor associated adipocytes presented a reduced size compared to adipose cells found farther away from the tumor’s invasive front (Fig. 6).

### hATT adipocytes showed significantly higher expression of versican, Adipo R1 and CD44, and significantly lower expression of adiponectin and perilipin, unlike hATN adipocytes

**Fig. 7:**
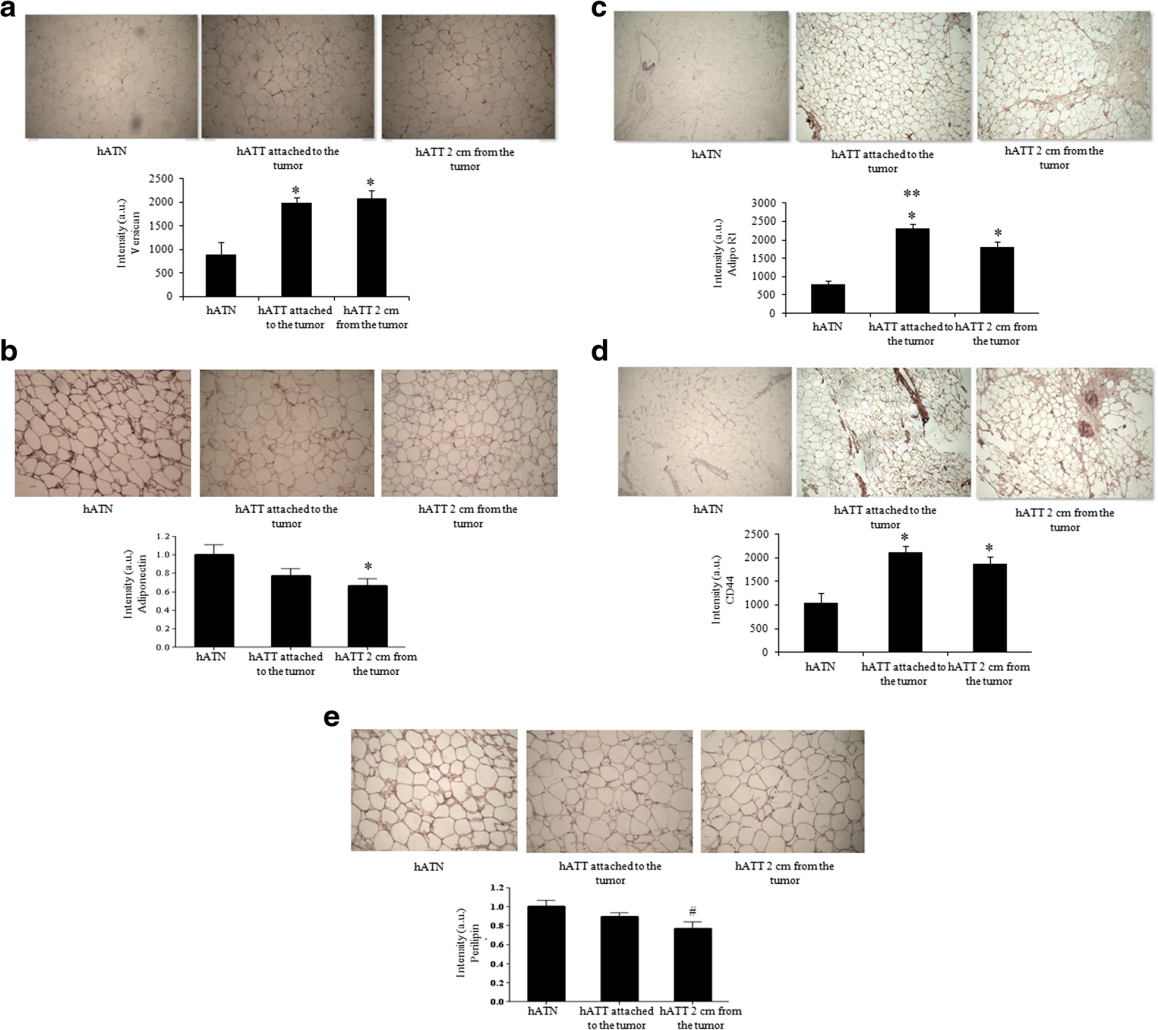
*Versican* (**a**)*, Adiponectin* (**b**)*, Adipo R1* (**c**)*, CD44* (**d**) *and Perilipin* (**e**) *expression in the different adipose tissues*. Versican, Adiponectin, Adipo R1, CD44 and Perilipin expression was evaluated by immunohistochemistry in serial cuts belonging to the same three tissue samples mentioned in Fig. 6. DAB staining quantification in the three tissue types was performed with Image J software (NIH). Histograms show mean ± SEM of four-five independent experiments. (a.u.: arbitrary units). *p < 0.05 hATT (both) *vs.* hATN; **p < 0.05 hATT (attached to tumor) *vs.* hATT 2 cm away from tumor; ^#^ *p* = 0.055 hATT (2 cm from the tumor) *vs.* hATN. Magnification: 100X

We evaluated expression of versican, adiponectin, Adipo R1, CD44, and perilipin expression of breast adipose tissues. Compared to hATN, we found increased expression of versican, Adipo R1 and CD44 both in hATT attached to the tumor as well as in hATT 2 cm from the tumor (Fig. 7a,c and d). In addition, hATT attached to the tumor showed significantly increased expression of Adipo R1 compared to hATT 2 cm from the tumor (Fig. 7c). Conversely, hATT adipocytes showed significantly lower expression of adiponectin and a decrease in perilipin, unlike hATN adipocytes (Fig. 7b and e). However, when we evaluated the levels of leptin and of its receptor, ObR, we didn’t find a significant difference between the samples tested (data not shown).

## Discussion

Stromal-epithelial interactions mediate the development and progression of breast cancer. Adipocytes are the predominant stromal cell type in breast tissue. We have recently shown that CMs from human adipose tissue explants of breast tumors differentially regulate the proliferation, adhesion and migration of breast cancer epithelial cell lines, compared to CMs from adipose tissue explants of normal breast samples [12]. In the present work we aimed to characterize factors that could be modified in tumor and non-tumor human breast epithelial cell lines, when incubated with hATT- or hATN-CMs, as possible candidate factors responsible of the observed biological effects. Specifically, we evaluated changes in the expression of versican, CD44, ADAMTS1 and Adipo R1. In addition, we evaluated the expression of versican and ADAMTS1 in hATN-CMs *vs* their expression in hATT-CMs. Finally, we observed changes in the phenotype of tumor-associated adipocytes compared to non tumor associated adipocytes; and evaluated with immunohistochemistry versican, adiponectin, Adipo R1, CD44, and perilipin expression in hATT and hATN slides. hATT-CMs significantly increased (in a dose dependent manner) tumor and non tumor epithelial cell proliferation. These results confirm previous observations already reported by our group, suggesting that soluble factors present in hATT-CMs stimulate breast epithelial cell proliferation in a dose-dependent manner, whether cells are tumor or not. These factors would appear not to be present (or present at lower concentrations) in hATN-CMs.

The identification of factors that might be modified in tumor and non-tumor breast epithelial cell lines when in contact with products secreted by adipose tissue from breast cancer samples, could allow studying the mechanisms by which adipose tissue might regulate tumor growth and progression. Previous works described versican as a component of the ECM; and it has been proposed that from this location it would help regulate cellular mechanisms such as proliferation, migration and angiogenesis. Nevertheless, recent experiments have also found an intracellular localization of versican, which implies new action mechanisms for this proteoglycan [28–30]. In accordance with these last reports, we observed that versican presents both a nuclear and a cytoplasmic distribution, although its nuclear values were higher. In addition, when tumor cells were incubated with hATT-CMs we observed an increase in total cell fluorescence compared to hATN- and control-CMs. Considering the nature of previous results, we propose that the observed increase in fluorescence intensity could be related to an increase in versican expression. We confirmed the observed increase of expression with a Western blot. Since versican presents 4 different isoforms, and it has been already seen that these isoforms have different functions [31], in future works we will analyze if there are changes in the proportion of these different isoforms, in addition to the increase in total expression.

Additionally, our results show that in breast cancer epithelial cells, hATT-CMs increase: 1) membrane glycoprotein CD44 (which plays a key role in cell adhesion as well as in cell migration); 2) metalloproteinase ADAMTS1 (which is capable of proteoglycan cleavage and ECM degradation); 3) adiponectin type 1 receptor (one of adiponectin receptors). In order to mechanistically evaluate the importance of these genes in cell proliferation, adhesion and migration, we will perform a loss- or gain-of-function in future experiments.

The identification of factors involved in the interaction between adipose and breast epithelial tissue could potentially allow us to regulate the expression of one or more of these factors in order to reverse tumor behavior.

Our results showed an increase of versican expression in CMs from hATT compared to CMs from hATN. This could indicate that verisican might be involved in the regulation of cell proliferation, adhesion and migration. To confirm, we performed an additional experiment in which we evaluated cell migration of MCF-7 and IBH-7 incubated with control-, hATN- and hATT-CMs pretreated (or not) with Chondroitinase ABC, an enzyme that degrades versican’s GAG chains. It has been reported that versican interacts with CD44, and this interaction depends on versican’s GAG chains [32, 33]. We found a loss of effect in cell migration values when hATT-CMs were pretreated with Chondroitinase ABC, thus strengthening the view that versican produced by adipocytes is involved at least in part in the biological effects seen in Pistone Creydt et al. [12].

ADAMTS1 gene is down-regulated in breast carcinomas with respect to non neoplastic mammary tissue [20]. Nevertheless it has been recently demonstrated that ADAMTS1 promotes breast tumor cell migration by regulating the release of semaphorin 3c from the ECM [34]. These apparently contradictory results could be due to the fact that ADAMTS1 presents two distinct active forms, an 87 kDa and a 65 kDa form, which have been describe to present different functions [35]. Although we didn’t find a significant difference in the levels of the 65 kDa form of this metalloproteinase in the hATT-CMs and hATN-CMs, a tendency to be increased in the hATN-CMs was seen. Since the 65 kDa form is mainly detached from the ECM and it has been reported to have anti-angiogenic properties, this could show a way in which a normal adipose microenvironment prevents angiogenesis in distant locations. Nonetheless, more samples need to be assessed to confirm these results. Furthermore, since an increase in ADAMTS1 expression not necessarily correlates with an increase in ADAMTS1 activity, we shall measure the activity of this peptidase in the different CMs and in the lysates of cells treated with the CMs.

In the last few years, several groups have demonstrated the importance of the dialogue that is established in the invasive front between tumor cells and stromal adipocytes [6, 7]. Tumor cells would be able to modify adipocyte phenotype, which in turn would stimulate the tumor’s aggressive behavior and local invasion [8]. We observed that the characteristics of the connective tissue were modified in response to the local invasion of tumor cells. Specifically, adipocytes associated to the tumor’s invasive front showed a reduced size compared to those found farther away (2 cm) and from adipocytes from normal breasts. In addition, we observed a significantly higher expression of versican, CD44 and Adipo R1, and significantly lower expression of adiponectin and perilipin, in hATT adipocytes compared to hATN adipocytes. We believe that the observed overexpression of Adipo R1 in both hATT tissue explants, and MCF-7 and IBH-7 cells incubated with hATT-CMs are an up-regulation compensatory mechanism as a consequence of the decreased levels of adiponectin secreted by peritumoral adipocytes. Serum adiponectin levels have been seen to significantly decrease in breast-cancer patients, while antiproliferative effects of adiponectin, as well as cell cycle inhibition and activation of apoptosis have been verified in most studies [36]. Our results are in line with these previous experiments, since they show that adiponectin levels are decreased in hATT tissue explants. In addition, we observed that perilipin (marker of mature adipocytes) is decreased and CD44 is increased in hATT versus hATN. This would mean that the adipocytes of the tumor microenvironment present a less differentiated state than the adipocytes of the normal microenvironment. This would indicate a loss in normal functions of mature adipocytes, such as energy storage, by others that could be favoring tumor development. These results begin to clarify early phenotypic and genotypic changes that undergo adipocytes that are in direct contact or farther away from the invasive tumor epithelium.

## Conclusions

We conclude that versican, a proteoglycan that is overexpressed in hATT-CMs compared to hATN-CMs, might be involved in the tumorogenic behavior observed in both cell lines employed. In addition, we may conclude that adipocytes from the tumor microenvironment show a less differentiated state than adipocytes from normal microenvironment. This would indicate a loss of normal functions in mature adipocytes (such as energy storage), in support of others that might favor tumor growth. The analysis and characterization of the factors that modify their expression, both in adipose tissue as well as epithelial cells, and the study of the involvement of these factors in the regulation of tumor progression, might allow developing new strategies to prevent and/or treat breast cancer.

## References

[1] Mueller MM, Fusenig NE (2004). Friends or foes – bipolar effects of the tumour stroma in cancer. Nat Rev Cancer.

[2] Wu Y, Kim JY, Zhou S, Smas CM (2008). Differential screening identifies transcripts with depot-dependent expression in white adipose tissues. BMC Genomics.

[3] Park J, Euhus DM, Scherer PE (2011). Paracrine and endocrine effects of adipose tissue on cancer development and progression. Endocr Rev.

[4] Finley DS, Calvert VS, Inokuchi J, Lau A, Narula N, Petricoin EF (2009). Periprostatic adipose tissue as a modulator of prostate cancer aggressiveness. J Urol.

[5] Landskroner-Eiger S, Park J, Israel D, Pollard JW, Scherer PE (2010). Morphogenesis of the developing mammary gland: stage-dependent impact of adipocytes. Dev Biol.

[6] Iyengar P, Espina V, Williams TW, Lin Y, Berry D, Jelicks LA (2005). Adipocyte-derived collagen VI affects early mammary tumor progression in vivo, demonstrating a critical interaction in the tumor/stroma microenvironment. J Clin Invest.

[7] Dirat B, Bochet L, Dabek M, Daviaud D, Dauvillier S, Majed B (2011). Cancer-associated adipocytes exhibit an activated phenotype and contribute to breast cancer invasion. Cancer Res.

[8] Wang YY, Lehuédé C, Laurent V, Dirat B, Dauvillier S, Bochet L (2012). Adipose tissue and breast epithelial cells: a dangerous dynamic duo in breast cancer. Cancer Lett.

[9] Schäffler A, Schölmerich J, Buechler C (2007). Mechanisms of disease: adipokines and breast cancer - endocrine and paracrine mechanisms that connect adiposity and breast cancer. Nat Clin Pract Endocrinol Metab.

[10] Walter M, Liang S, Ghosh S, Hornsby PJ, Li R (2009). Interleukin 6 secreted from adipose stromal cells promotes migration and invasion of breast cancer cells. Oncogene.

[11] Pistone Creydt V, Sacca PA, Tesone AJ, Vidal L, Calvo JC (2010). Adipocyte differentiation influences the proliferation and migration of normal and tumoral breast epithelial cells. Mol Med Report.

[12] Pistone Creydt V, Fletcher SJ, Giudice J, Bruzzone A, Chasseing NA, Gonzalez EG (2013). Human adipose tissue from normal and tumoral breast regulates the behavior of mammary epithelial cells. Clin Transl Oncol.

[13] Touab M, Villena J, Barranco C, Arumí-Uría M, Bassols A (2002). Versican is differentially expressed in human melanoma and may play a role in tumor development. Am J Pathol.

[14] Skandalis SS, Labropoulou VT, Ravazoula P, Likaki-Karatza E, Dobra K, Kalofonos HP (2011). Versican but not decorin accumulation is related to malignancy in mammographically detected high density and malignant-appearing microcalcifications in non-palpable breast carcinomas. BMC Cancer.

[15] Hernández D, Miquel-Serra L, Docampo MJ, Marco-Ramell A, Bassols A (2011). Role of versican V0/V1 and CD44 in the regulation of human melanoma cell behavior. Int J Mol Med.

[16] Al-Hajj M, Wicha MS, Benito-Hernández A, Morrison SJ, Clarke MF (2003). Prospective identification of tumorigenic breast cancer cells. Proc Natl Acad Sci U S A.

[17] Hamid AA, Idrus RBH, Saim AB, Sathappan S, Chua KH (2012). Characterization of human adipose-derived stem cells and expression of chondrogenic genes during induction of cartilage differentiation. Clinics.

[18] Ween MP, Oehler MK, Ricciardelli C (2011). Role of versican, hyaluronan and CD44 in ovarian cancer metastasis. Int J Mol Sci.

[19] McClements L, Yakkundi A, Papaspyropoulos A, Harrison H, Ablett MP, Jithesh PV (2013). Targeting treatment-resistant breast cancer stem cells with FKBPL and its peptide derivative, AD-01, via the CD44 pathway. Clin Cancer Res.

[20] Porter S, Scott SD, Sassoon EM, Williams MR, Jones JL, Girling AC (2004). Dysregulated expression of adamalysin-thrombospondin genes in human breast carcinoma. Clin Cancer Res.

[21] Gonzalez-Perez RR, Lanier V, Newman G (2013). Leptin’s pro-angiogenic signature in breast cancer. Cancers.

[22] Obeid S, Hebbard L (2012). Role of adiponectin and its receptors in cancer. Cancer Biol Med.

[23] Housa D, Housová J, Vernerová Z, Haluzík M (2006). Adipocytokines and cancer. Physiol Res.

[24] Jeong YJ, Bong JG, Park SH, Choi JH, Oh HK (2011). Expression of leptin, leptin receptor, adiponectin, and adiponectin receptor in ductal carcinoma in situ and invasive breast cancer. J Breast Cancer.

[25] Lyu Y, Su X, Deng J, Liu S, Zou L, Zhao X, Wei S, Geng B, Xu G (2015). Defective differentiation of adipose precursor cells from lipodystrophic mice lacking perilipin 1. PLoS One.

[26] Vázquez SM, Mladovan A, Garbovesky C, Baldi A, Lüthy IA (2004). Three novel hormone-responsive cell lines derived from primary human breast carcinomas: functional characterization. J Cell Physiol.

[27] Bruzzone A, Vanzulli SI, Soldati R, Giulianelli S, Lanari C, Lüthy IA (2009). Novel human breast cancer cell lines IBH-4, IBH-6, and IBH-7 growing in nude mice. J Cell Physiol.

[28] Carthy JM, Abraham T, Meredith AJ, Boroomand S, McManus BM (2015). Versican localizes to the nucleus in proliferating mesenchymal cells. Cardiovasc Pathol.

[29] Rahmani M, Wong BW, Ang L, Cheung CC, Carthy JM, Walinski H (2006). Versican: signaling to transcriptional control pathways. Can J Physiol Pharmacol.

[30] Carthy JM, Boroomand S, McManus BM (2012). Versican and CD44 in in vitro valvular interstitial cell injury and repair. Cardiovasc Pathol.

[31] Sheng W, Wang G, Wang Y, Liang J, Wen J, Zheng PS (2005). The roles of versican V1 and V2 isoforms in cell proliferation and apoptosis. Mol Biol Cell.

[32] Kawashima H, Hirose M, Hirose J, Nagakubo D, Plaas AH, Miyasaka M (2000). Binding of a large chondroitin sulfate/dermatan sulfate proteoglycan, versican, to L-selectin, P-selectin, and CD44. J Biol Chem.

[33] Wu YJ, La Pierre DP, Wu J, Yee AJ, Yang BB (2005). The interaction of versican with its binding partners. Cell Res.

[34] Esselens C, Malapeira J, Colomé N, Casal C, Rodríguez-Manzaneque JC, Canals F (2010). The cleavage of semaphorin 3C induced by ADAMTS1 promotes cell migration. J Biol Chem.

[35] Rodríguez-Manzaneque JC, Milchanowski AB, Erick K, Dufour EK, Leduc R, Iruela-Arispe ML (2000). Characterization of METH-1/ADAMTS1 processing reveals two distinct active forms. J Biol Chem.

[36] Kang JH, Lee YY, Yu BY, Yang BS, Cho KH, Yoon DK, Roh YK (2005). Adiponectin induces growth arrest and apoptosis of MDA-MB-231 breast cancer cell. Arch Pharm Res.

